# “A beautiful chaos”: a qualitative study on exploring the role of international communities of practice in knowledge translation for sexual and reproductive health and rights

**DOI:** 10.1080/26410397.2026.2714691

**Published:** 2026-08-17

**Authors:** Noa E.M. Kolpa, Nicole Moran, Joyce L. Browne, on behalf of the Community of Practice Study Group

**Affiliations:** a Student assistant, Department of Global Public Health and Bioethics, Julius Center for Health Sciences and Primary Care, University Medical Center Utrecht, Utrecht University, The Netherlands.; b Sexual and Reproductive Health and Rights Advisor, Sexual and Reproductive Health and Rights Department, Koninklijk Instituut voor de Tropen, The Netherlands; c Associate Professor Global Health, Department of Global Public Health and Bioethics, Julius Center for Health Sciences and Primary Care, University Medical Center Utrecht, Utrecht University, The Netherlands; Utrecht Center for Global Challenges, Faculty of Law, Economics and Governance, Utrecht University, Utrecht, The Netherlands

**Keywords:** sexual and reproductive health and rights, knowledge translation, community of practice, contextual factors, implementation science

## Abstract

Persistent knowledge-to-action gaps in sexual and reproductive health and rights (SRHR) limit the translation of evidence into policy and practice. Knowledge platform Share-Net International (SNI) uses Communities of Practice (CoPs) to support knowledge exchange, yet little is known about how CoPs facilitate knowledge translation. This study examined how CoPs across SNI’s networks translate SRHR knowledge into action and identified contextual factors shaping these processes. A transdisciplinary qualitative study (October 2024–January 2025) was conducted across eight SNI hubs. CoP members and facilitators with at least three months of involvement were purposely sampled. Data were transcribed verbatim and analysed inductively using thematic analysis in Atlas.ti guided by the knowledge-to-action framework and triangulated through member-checking. Outcomes included knowledge translation processes, CoP functioning, and contextual factors. CoPs create context-specific knowledge, facilitate learning spaces, and produce knowledge products. However, significant gaps were observed in dissemination planning and M&E, limiting the ability to assess impact. Political resistance, conservative norms, power dynamics, and crises were challenges to effective functioning, while supportive policies, activism, and cross-hub learning created opportunities. Strategies to strengthen CoP effectiveness were proposed. CoPs hold strong potential to advance knowledge translation for SRHR, but their effectiveness depends on structured dissemination, learning-oriented M&E, and approaches responsive to local contexts. As CoPs operate within power structures, their ability to contribute to equitable SRHR outcomes is shaped by the environments in which they function. Strengthening facilitation, resources, and cross-hub collaboration may enhance effectiveness, and longitudinal research is recommended to assess sustained policy and practice change. DOI: 10.1080/26410397.2026.2714691

## Plain language summary

Access to sexual and reproductive health and rights (SRHR) is still unequal in many parts of the world, despite progress in recent years. One reason is that knowledge from research doesn’t always reach the people who make policies or deliver services. Share-Net International (SNI) tries to bridge this gap by bringing people together in Communities of Practice (CoPs), groups where individuals share knowledge, ideas, and work together on SRHR topics. This study looked at how these CoPs operate in different countries and what helps or hinders their efforts to make SRHR knowledge useful in real-world settings. Researchers interviewed and spoke with people involved in these groups to understand their experiences. They found that CoPs are valuable spaces for learning and creating useful tools. However, they face several challenges, such as limited resources, low participation, and difficulty measuring their impact. Broader issues, like political resistance, unequal power dynamics, and crises, can also make their work harder. On the other hand, supportive policies and social movements can boost their efforts. In short, while CoPs have real potential to improve SRHR knowledge and action, they need flexible strategies, strong collaboration, and ongoing support to make a lasting difference.

## Résumé

Les écarts persistants dans le passage de la connaissance à l’action dans le domaine de la santé et des droits sexuels et reproductifs limitent la mise en pratique des données disponibles dans les politiques et les pratiques. La plateforme de connaissances Share-Net International (SNI) utilise les communautés de pratique pour favoriser l’échange de connaissances, mais on sait peu de choses à propos de la manière dont ces communautés facilitent la traduction des connaissances dans la pratique. Cette étude a examiné comment les communautés de pratique des différents pôles de la SNI appliquent concrètement les connaissances en santé et droits sexuels et reproductifs, et elle a identifié les facteurs contextuels qui façonnent ces processus. Une étude qualitative transdisciplinaire (octobre 2024-janvier 2025) a été menée dans huit pôles de la SNI. Les membres et les animateurs des communautés de pratique ayant au moins trois mois d’activité ont été sélectionnés de manière délibérée. Les données ont été transcrites intégralement et étudiées inductivement par analyse thématique dans Atlas.ti, en s’appuyant sur le cadre de la connaissance à l’action et en triangulant les résultats par vérification auprès des participants. Les résultats ont porté sur les processus de traduction des connaissances, le fonctionnement des communautés de pratique et les facteurs contextuels. Les communautés de pratique créent des connaissances adaptées au contexte, animent des espaces d’apprentissage et produisent des ressources du savoir. Néanmoins, des lacunes importantes ont été observées dans la planification de la diffusion et le suivi-évaluation, limitant ainsi la capacité d’évaluer l’impact. La résistance politique, les normes conservatrices, les dynamiques de pouvoir et les crises ont constitué des obstacles à leur bon fonctionnement, alors que les politiques de soutien, l’activisme et l’apprentissage dans plusieurs pôles ont créé des opportunités. Des stratégies destinées à renforcer l’efficacité des communautés de pratique ont été proposées. Les communautés de pratique possèdent un fort potentiel pour faire progresser la traduction des connaissances en matière de santé et de droits sexuels et reproductifs, mais leur efficacité dépend d’une diffusion structurée, d’un suivi-évaluation axé sur l’apprentissage et d’approches adaptées aux contextes locaux. Puisque les communautés de pratique opèrent dans le cadre de structures de pouvoir, leur capacité de contribution à des résultats équitables en matière de santé et droits sexuels et reproductifs est façonnée par les environnements dans lesquels elles évoluent. Renforcer la facilitation, les ressources et la collaboration entre les pôles pourrait améliorer leur efficacité, et une recherche longitudinale est recommandée pour évaluer les changements durables apportés aux politiques et aux pratiques.

## Resumen

Las brechas persistentes entre el conocimiento y la acción en salud y derechos sexuales y reproductivos (SDSR) limitan traducir la evidencia en políticas y prácticas. La plataforma de conocimiento *Share-Net International* (SNI) utiliza Comunidades de Práctica (CdP) para apoyar el intercambio de conocimientos; sin embargo, no se sabe mucho sobre la manera en que las CdP facilitan la traducción de conocimiento. Este estudio examinó la manera en que las CdP en diferentes centros de SNI traducen en acción su conocimiento sobre SDSR e identificó los factores contextuales que moldean estos procesos. Se realizó un estudio cualitativo transdisciplinario (entre octubre de 2024 y enero de 2025) en ocho centros de SNI. Los miembros y facilitadores de las CdP con un mínimo de tres meses de participación fueron seleccionados mediante muestreo intencional. Los datos fueron transcritos textualmente y analizados inductivamente utilizando análisis temático en Atlas.ti guiado por el Marco del Conocimiento a la Acción y triangulado por medio de la verificación de miembros. Los resultados abarcaron procesos de traducción de conocimiento, funcionamiento de las CdP y factores contextuales. Las CdP crean conocimiento específico al contexto, facilitan espacios de aprendizaje y producen productos de conocimiento. Sin embargo, se observaron brechas significativas en la planificación de difusión y en M&E, que limitan la capacidad para evaluar el impacto. Resistencia política, normas conservadoras, dinámicas de poder y crisis eran retos al funcionamiento eficaz, mientras que las políticas solidarias, el activismo y el aprendizaje entre centros crearon oportunidades. Se propusieron estrategias para fortalecer la eficacia de las CdP, las cuales tienen un gran potencial de promover la traducción de conocimiento sobre SDSR, pero su eficacia depende de una difusión estructurada, de M&E orientados al aprendizaje y de enfoques adaptados a los contextos locales. Dado que las CdP operan dentro de estructuras de poder, su capacidad para contribuir a resultados equitativos de SDSR se ve afectada por los entornos donde funcionan. Su eficacia podría mejorar al reforzar la facilitación, los recursos y la colaboración entre centros, y se recomienda realizar investigaciones longitudinales para evaluar el cambio sostenido en las políticas y prácticas.

## Introduction

Although substantial progress has been made in the last decades in sexual and reproductive health and rights (SRHR), challenges to universal access persist.^1,2^ Achieving SRHR for all means promoting overall sexual and reproductive well-being by ensuring safe, consensual, and pleasurable experiences, along with the ability and freedom to choose if, when, and how to reproduce, while respecting everyone's rights and ensuring good-quality health services.^1^ Yet, significant knowledge-to-action (KtA) gaps persist, which arise from multiple factors, including limited access to evidence-based SRHR information, insufficient dissemination to practitioners and policymakers, and contextual barriers such as sociocultural norms, gender inequalities, political resistance and resource constraints.^3–7^ These limitations hinder the translation of scientific evidence and community-generated insights into effective policy and practice. Populations disproportionately affected include adolescents, young people, women and girls in rural or marginalised settings, and communities living under restrictive sociopolitical conditions.^8,9^

One of the approaches to improving SRHR is effective knowledge management.^6,10^ Knowledge management involves systematically locating, storing, retrieving, disseminating, adapting, and using knowledge to ensure that it is effectively available and applied.^11^ An important pillar in knowledge management is knowledge translation (KT). Various definitions of KT exist, but for this article, the definition from the Canadian Institutes of Health Research is adopted: “*Knowledge translation is a dynamic and iterative process that involves the synthesis, dissemination, exchange, and ethically-sound application of knowledge to enhance health, deliver more effective health services and products, and strengthen the healthcare system*.”^12^ Without effective KT, evidence often remains underutilised within NGOs, academia, and research institutions, limiting its influence on policy and practice.

Addressing the KtA gap in SRHR requires effective mechanisms and strategies to translate evidence into policy and practice. Knowledge platforms may play a critical role in this process by fostering collaboration among diverse stakeholders.^13,14^ One example of such a platform is Share-Net International (SNI), a global knowledge platform for SRHR funded by the Dutch Ministry of Foreign Affairs. Established in 2001 in the Netherlands, Share-Net has grown into an international network consisting of over 3500 members with hubs in seven countries: Bangladesh, Burkina Faso, Burundi, Colombia, Ethiopia, Jordan, and the Netherlands. SNI aims to connect research, policy, and practice through strategic knowledge generation, translation, dissemination, networking, and the promotion of knowledge use. By integrating global expertise with localised insights, SNI seeks to improve SRHR policies and practices, ensuring that “*all people have freedom of choice, are able to make informed decisions, can take action on their sexual and reproductive health, and have access to quality reproductive health needs and rights*.”^15^

One of the strategies employed by SNI to achieve this mission is using Communities of Practice (CoPs). The term “CoP” describes a “*group of individuals with shared interests that come together – in person or virtually – to tell stories, share and discuss problems and opportunities, discuss best practices, and review lessons learned*.”^16^ First coined in 1998 by Etienne Wenger, CoPs were identified as a strategy for companies to improve workplace learning, exchange and increase productivity.^17^ The use of CoPs to improve learning and promotion of best practices has since expanded across a number of sectors, including in public health,^18,19^ as an effective mechanism for sharing tacit knowledge.^20^ While first understood to support learning between colleagues within the same organisation, the concept of a CoP has expanded over recent decades to include other people working in the same field of interest, who experience the same issues and knowledge gaps.^20^ This means that CoPs can be local, national, regional or global. Existing research highlights that CoPs can contribute to positive changes in implementing best practices and improving practice (Box 1).^18–20^

**Box 1. Community of Practice (CoP)**

A Community of Practice (CoP) is a “group of individuals with shared interests that come together - in person or virtually - to tell stories, share and discuss problems and opportunities, discuss best practices, and review lessons learned”.^15^

Considering the knowledge-to-action gap that exists in the field of SRHR, and the success of CoPs in the public health sector to support linking and learning of experts to address knowledge and implementation gaps, CoPs have been used as a vehicle within the SNI network for over 10 years to support members in bridging this gap. Between 2020 and 2024, the number of active CoPs across the SNI network grew from 25 to 44. Through moderated CoPs, SNI members contribute to, discuss, share, use, and have access to evidence and knowledge-products that are relevant to their specific areas of work for improved SRHR policies and practices. Knowledge translation is the most common process within these CoPs, but they also play a role in other aspects of knowledge management.^21,22^

The thematic focus of CoPs across SNI are wide-ranging and include topics such as preventing child marriage, the intersection between SRHR and the climate crisis, access to contraception and abortion, and engaging men and boys.^21,22^ Members of CoPs are diverse and can include university staff and researchers, people who work in NGOs, government representatives, young people and students, and activists. CoPs vary widely in lifespan, size, structure, and setting, ranging from small local groups to extensive international networks, and from highly organised to more informal, spontaneous formations.^23,24^ They may be established for a short period of time to address a specific knowledge gap or activity, or they can exist for many years, with active members changing over their lifespan.

The diversity between CoPs and the uniqueness of each one makes them flexible to the learning needs of the members, but this also makes it difficult to assess their long-term impact. CoPs were not required to report on their activities as a condition of receiving funding, but the Secretariat of each country hub was responsible for developing an annual report on all their activities, including the CoP activities of that year. Over the past two decades, SNI CoPs have contributed to SRHR knowledge translation through the development of knowledge products, facilitation of cross-country learning, and support for evidence-informed advocacy, but systematic evaluation of long-term impact remains limited.

Although CoPs are increasingly used as an approach to facilitate KT, evidence on how they support KT and how their functioning is shaped by different contexts remains limited.^18–20^ Existing literature on KT in SRHR has predominantly focused on rapid KT approaches and scoping reviews of KT strategies.^4,25,26^ While emerging evidence has begun to identify mechanisms through which CoPs facilitate KT in health settings, limited attention has been given to how these mechanisms operate within SRHR-focused CoPs.^18–20^ In particular, little is known about how contextual factors enable or constrain KT within international knowledge platforms such as SNI.

The purpose of this study is therefore to explore how the CoPs across the Share-Net International network facilitate the translation of knowledge into policy and practice within the field of SRHR, and what contextual factors influence these efforts.

## Methods

### Study design, setting and period

This transdisciplinary study employed a qualitative design, with semi-structured interviews and focus group discussions (FGDs) conducted across Share-Net networks between October 2024 and January 2025. The combination of individual interviews and FGDs was chosen to enable both in-depth exploration of individual perspectives and collective reflection on shared experiences. While interviews allowed participants to provide detailed insights into their roles, experiences, and perceptions of the CoPs functioning, FGDs facilitated interaction between participants, enabling the validation, comparison, and expansion of emerging findings, as well as the co-creation of interpretations and potential strategies. All interviews and FGDs were conducted online, with participants located across the eight Share-Net networks: Bangladesh, Burkina Faso, Burundi, Colombia, Ethiopia, Jordan, the Netherlands, and Share-Net International. These networks represent the full scope of Share-Net’s global platform, allowing insights across diverse contexts in which CoPs operate and knowledge translation takes place.

### Characteristics of the participants

Eligible participants included CoP members and facilitators from all eight Share-Net networks. Facilitators, who are Share-Net staff, provide both logistical and content support for CoPs, while CoP members voluntarily drive the work of the CoPs. To be eligible for inclusion, participants needed at least three months of active involvement in a CoP, a background in SRHR, and sufficient English language proficiency.

### Recruitment of the participants

Facilitators were contacted via email to schedule interviews and identify potential CoP members through snowball sampling. Members were purposefully selected based on their background, involvement duration, and CoP theme to ensure diverse perspectives, then invited via email to participate. The study aimed to include at least one facilitator and one CoP member from each network, resulting in a minimum target of sixteen participants. This sampling strategy was intended to ensure representation across networks while supporting the achievement of data saturation. The final sample comprised 15 interview participants. Participants varied in their level of engagement, ranging from involvement in one to eight CoPs, with a total of 32 CoPs represented. All interview participants were invited to participate in the FGDs; two FGDs were conducted, with three participants in the first and five in the second. For the seven interview participants who were unable to attend, an email-based member-checking process was conducted.

### Data collection

Interviews were conducted individually online via Microsoft Teams in English by one researcher (NK), lasting 35–75 minutes. A semi-structured interview guide was used, covering: (1) the functioning and structure of CoPs, (2) knowledge translation processes, and (3) contextual factors influencing CoP activities (e.g. resources, political environment, member engagement, communication, and organisational structures).^27^ The full interview guide is provided in Supplementary File 1.

Two online FGDs were conducted using Microsoft Teams and Mural (a visual collaboration tool), and lasted two hours, with one researcher acting as the moderator (NK) and a second researcher assisting (JB or JA). FGDs explored: (1) preliminary findings from the interviews, (2) key challenges experienced by CoPs (resources, dissemination, monitoring and evaluation, and member engagement), and (3) potential strategies to address contextual barriers. The FGD guide is included in Supplementary File 2. For those unable to attend the FGD, an additional email-based member-checking process was conducted, allowing them to review transcripts and coding. They could request corrections or flag information they felt could compromise their safety; such data were removed prior to analysis.

### Data analysis

Thematic analysis was used to analyse the data from both interviews and the FGDs. Audio recordings were transcribed verbatim and coded using Atlas.ti (developed by ATLAS.ti Scientific Software Development GmbH, Berlin, Germany), guided by Braun et al.'s thematic analysis approach.^28^ Data analysis followed an iterative, multi-stage process. First, during familiarisation, the primary researcher (NK) read and reread all transcripts, taking notes on emerging patterns related to CoP functioning, KT processes, contextual influences, and participant experiences. This was followed by initial coding, in which open coding was applied line-by-line to all transcripts. Codes captured segments relating to: (1) CoP functioning, (2) knowledge translation processes, (3) contextual influences, and (4) challenges and opportunities. Descriptive codes on participant roles and CoP characteristics were generated inductively for the “Participant and CoP characteristics” section. While theme development was guided by the research questions, codes related to KT processes were mapped to phases of the KtA Framework, which served as an analytical scaffold for organising data on knowledge creation, adaptation, dissemination, implementation, and evaluation. The KtA framework shows how knowledge is created and then applied through an iterative Action Cycle. The cycle includes identifying a problem and the know–do gap, adapting knowledge to context, assessing barriers and facilitators, implementing tailored interventions, monitoring knowledge use, evaluating outcomes, and sustaining knowledge use.^27^

Next, axial coding was conducted by comparing codes within and across transcripts to identify similarities, differences, and patterns. Related codes were grouped into broader categories by examining conceptual links, clustering codes with similar meanings, and exploring relationships between inductive codes and the KtA-informed deductive codes. FGD data were integrated by merging overlapping categories and adding challenge-specific codes (resources, dissemination, M&E, member engagement). To support analytic transparency, recurring code groups were organised into tables (e.g. challenges, opportunities, contextual factors, strategies).

Finally, categories were iteratively refined into themes by examining how groups of codes captured shared concepts and how they contributed to answering the research questions. The resulting themes included participant and CoP characteristics, KT practices, internal challenges and opportunities, contextual factors, and strategies to address challenges. Data extracts within each theme were reviewed to ensure internal coherence and clear conceptual boundaries between themes. To strengthen the credibility of the analysis, researcher triangulation was undertaken: a second researcher (NM) independently coded one interview and discrepancies were discussed within the research team (NK, NM, JB), leading to refinements of the coding framework and improved shared interpretation. In addition, participant feedback, including member-checking feedback and input from a Share-Net International feedback session, was incorporated into the refinement of themes and interpretation of findings.

### Ethical considerations

An ethical waiver was given by the ethics committee of KIT Institute on 3 October 2024, because participants were interviewed within their professional capacity. Participants were informed about the study’s objectives, and they were requested to provide written consent for audio recording and data processing. They were also encouraged to ask questions or raise concerns. Moreover, they were assured that their participation was voluntary, and that if they wanted to withdraw from the research, they could do so at any point. The informed consent process was reiterated at the start of each interview and FGD to ensure participants’ ongoing understanding and agreement.

All data were securely stored on a protected server, accessible only to the research team. To ensure confidentiality, all data were pseudo-anonymised by assigning a unique code to each participant. Additionally, background characteristics were excluded in the presentation of results, as this could inadvertently facilitate the identification of participants based on unique attribute combinations.

The research team's positionality inevitably influenced the data's analysis and interpretation. The primary researchers, based in the Netherlands, have backgrounds in biomedical science, medicine, epidemiology, global health, and gender studies. To address this, we adopted a reflexive approach, actively challenging our assumptions and engaging in critical self-reflection throughout the research process. This was supported by ongoing discussions with the wider research team which had additional disciplinary backgrounds, including implementation science, science and technology studies, feedback from participants and Share-Net members, and the invitation of participants to contribute as co-authors. These transdisciplinary collaborative efforts ensured that the findings remained grounded in the lived realities of CoP members and the broader SRHR context.

## Results

The results below are presented thematically, combining insights from FGDs and interviews. First, participant and CoP characteristics will be discussed, followed by KT process engaged in by the CoPs, internal challenges and opportunities of the CoPs, contextual factors that affect the CoPs’ functioning, and finally, identified strategies to address challenges. For clarity in the sections below, we briefly reiterate the definition of a CoP used in this paper: a CoP is “a group of individuals with shared interests that come together – in person or virtually – to tell stories, share and discuss problems and opportunities, discuss best practices, and review lessons learned.”^16^

### Participant characteristics

Participants' characteristics are summarised in Table 1. Participants had varying levels of involvement in their CoPs, ranging from less than a year to as long as thirteen years, with most having participated for a few years. The roles of CoP members within the CoPs were diverse and included leadership positions as well as more supportive roles. Participants also brought a wide range of professional backgrounds to their CoP involvement, for example as a medical doctor, non-governmental organisation (NGO) staff, academic, student, SRHR advisor, and Share-Net staff member.

**Table 1.** Participant characteristics

Participant ID	Role (Staff / Member)*	Years in CoP (1–3 years, 4–5 years, 6 + years)	Level of involvement (low/medium/high)	Member of the following CoP(s)
P01	Staff	5+	High	Child marriage, climate and SRHR, infertility, menstrual health, CSE, SRHR in fragile settings, GBV, SRHR and access to information
P02	Staff	1–3	High	SRHR for adolescence and youth, media & SRHR, family planning, menstrual health, HIV, diversity and inclusion.
P03	Staff	4–5	High	Safe abortion, sexuality and aging
P04	Staff	4–5	High	Media & SRHR, SRHR for adolescence and youth, gender-based violence
P05	Staff	1–3	High	SRH and mental health, maternal health, SRH for adolescence and youth.
P06	Staff	1–3	High	Sexual pleasure, contraception and abortion
P07	Staff	1–3	Hgh	Engaging men and boys, youth and SRHR, gender-based violence
P08	Member	4–5	Medium	Engaging men and boys
P09	Member	6+	Medium	Sexual pleasure, menstrual health, contraception and abortion
P10	Member	4–5	High	SRH for adolescence and youth
P11	Member	1–3	High	SRHR and access to information
P12	Member	4–5	Medium	Gender-based violence
P13	Member	6+	High	SRHR for adolescence and youth
P14	Member	1–3	High	Gender-based violence
P15	Member	4–5	High	SRHR for adolescence and youth

“Staff” can be understood as paid Share-Net secretariat colleagues who facilitate CoPs. “Member” can be understood as an individual or representative of an organisation who is a member of the Share-Net network and who participates in a CoP either on behalf of their organisation or on a voluntary individual basis.

The interviewees participated in CoPs addressing a wide range of themes related to SRH(R). These included youth and adolescence, sexual pleasure, contraception, (safe) abortion, sexuality and aging, menstrual health, media, family planning, HIV, diversity and inclusion, roles and needs of men and boys in SRHR, the role of adolescents and parents, the role of religious leaders, gender-based violence (GBV), infertility, mental health, maternal health, use of technology, child marriage, SRHR in fragile settings, climate change and SRHR, access to SRHR information, regional issues, and comprehensive sexuality education. Among these, GBV and youth and adolescence were the most frequently mentioned.

### CoP characteristics

The work processes of CoPs varied across countries, themes, and over time. Some CoPs followed structured approaches, with annual plans, blueprints, or proposals, while others operated more flexibly through informal, occasional meetings. Their longevity also varied, with some lasting less than a year and others continuing longer, often depending on their established goals. One participant described CoPs as a “beautiful chaos”, highlighting their organic and adaptable nature.

All CoPs primarily relied on small-scale funding from the Dutch Ministry of Foreign Affairs, which was allocated for knowledge product development and learning activities and the salary of the CoP facilitators, who were always Share-Net secretariat staff. In many cases, participants noted that additional funding sources were either not sought or unknown. When other funding was secured, it typically came through co-creation initiatives that CoP members engaged in.

### Knowledge translation processes engaged in by the CoPs

The following analysis draws on the KtA framework, each subheading reflecting a phase of the KT process as conceptualised within this framework.^27^ Supplementary File 3 provides a table of internal CoP challenges and opportunities, including codes, themes, and descriptions.

#### Identifying main problems: how CoP members collaboratively determined the key issues they sought to address

CoPs typically began with internal discussions among members to identify the key issue they wanted to address.

“*And what we do is like a big brainstorm with all the CoP members. Once we've done that, we prioritise the ideas, we seek common interest*.” (P03)

At the outset, members were asked to express their views on priority thematic areas based on their programming and interests, which served as the foundation for developing each community of practice. Over time, these focus areas often evolved in response to changes within the broader SRHR landscape. For example, one CoP was described as “*very crosscutting and touching various other thematics like immunization, nutrition, child protection, etc.”* (P02). This CoP was able to continuously adapt its scope in response to the shifts in the SRHR landscape. Drawing on their professional experience and responding to regional, national, or global developments, members would collaboratively define and refine their areas of focus throughout the process.

#### Knowledge sources: diverse sources of information informed CoP activities

Participants relied on multiple sources of knowledge for their CoP’s work. These included the experiences of Share-Net members, local and social media, government, NGOs and other organisations, as well as scientific journals and grey literature. Local organisations, particularly community-based groups, were highlighted as especially valuable because of their proximity to on-the-ground realities. As one participant stated:

“*We work with a lot of organisations … a lot of them are community-based organisations … actually working in the territory or in the field … matched up with the bigger organisations that may be a little bit further from what is actually happening in reality*.” (P03)

Another participant emphasised that the diversity of organisational members enriched the CoPs’ knowledge base:

“*Each of them are sources of knowledge … each one of the people that are there are a very important source of knowledge about the particularities and the context that different communities in different spaces are facing*.” (P12)

The specific CoP objectives and the member composition of the CoP shaped which sources of information were necessary for the CoP but across contexts, CoPs relied mostly on the knowledge and experience of their existing members to identify knowledge gaps and brainstorm on solutions.

#### Knowledge activities: a range of knowledge management and KT activities undertaken by the CoPs

One type of knowledge management (knowledge creation) and two types of KT activities (learning space and knowledge products) were identified. Participants described their activities as including both knowledge management, such as generating and organising knowledge, and knowledge translation activities, such as sharing and applying it. Knowledge creation primarily involved research (for example, a study on experiences of menstrual health in the workplace), whereas learning spaces facilitated engagement through workshops, training sessions, and political debates. As one participant described:

“*When we have a meeting, workshop, we really learn … we are still exploring, we are still discovering new knowledge and trying to translate them into knowledge that can be accessible … especially in the rural areas and especially [for] young people*.” (P14)

Knowledge products, such as toolkits, infographics, media posts, podcasts, videos, games, policy briefs, and campaigns, served as practical tools for sharing and applying knowledge. One participant detailed a comprehensive set of activities undertaken for a gender-based violence initiative, including sharing laws, showcasing service provision experiences, and developing multilingual online campaigns:

“*We made [an] online campaign to inform people about gender based violence … and also to share, to translate all this information into local language … we try also to use some strategies of communication to reach those people*.” (P14)

#### Implementation and dissemination: channels and strategies used by CoPs to share and apply knowledge products

Participants recognised a range of channels for implementing and disseminating knowledge products. These included internal networks (CoP members, within Share-Net, Share-Net events), external collaborations (partner organisations, stakeholder meetings, external events), mass media (social media, websites, newsletters, physical paper, radio, and billboards) and community-based approaches (schools, universities, and communities). Training facilitators and users to apply knowledge products was also mentioned as a strategy. For example, one participant described how the CoP used billboards and radio to reach young people:

“*In the marketplace … big billboards have information about contraceptives and HIV … And … the main things that we use to disseminate … is radio … we share different SRH information by doing drama, storytelling … and also [provide] training for the community, religious leaders and also for schools*.” (P11)

However, some participants noted that dissemination was often treated as a later-stage activity rather than being strategically planned alongside the development of knowledge products, which limited the effectiveness and reach of dissemination efforts. This challenge was echoed by multiple participants, reflecting a broader pattern within SNI. As one participant explained:

“*When we're designing the product, we do not think about how to share it. So we create the product and then we go, what then?*” (P03)

#### Monitoring and evaluation (M&E): how CoPs monitored and evaluated their activities and the existing gaps

Evaluation was conducted through internal discussions and feedback given by members of the CoP who were involved in the activity. Some CoPs also made use of a post-activity survey where participants gave feedback which was used to evaluate the impact of an activity. When external stakeholders, such as NGOs, researchers, or thematic experts, were involved, online debriefing meetings were organised to reflect on the activity and give additional feedback.

In addition to these CoP-level M&E efforts, the Share-Net secretariats also conducted M&E for overall CoP activities. This included social media analysis by Share-Net communications staff and analysis of data collected by Share-Net’s M&E staff for the annual report which incorporated indicators related to CoP activities.^29^

Participants noted that clear M&E strategies were often lacking, leading to uncertainty about the reach and impact of knowledge products. Several participants described the absence of systems to track how knowledge products were disseminated, which target groups were reached, and whether end users provided feedback or used the information in practice. One participant emphasised the need for better tracking mechanisms to demonstrate progress:

“*We are doing so much, a lot of work. But unfortunately, due to lack of tracking this impact, we cannot show our progress*.” (P01)

Others highlighted the importance of routine documentation for monitoring implementation processes and outcomes. Without systematic documentation, participants explained that there was often little information available on how initiatives unfolded, who participated, and what effects were achieved. As one FGD participant reflected:

“*I think sometimes something is done and then … the completion of an activity or an event is often seen as OK, now this is done. And not enough is done to capture … what was the process … how many people were involved … we just need to get into the habit of doing very basic standard documentation*.” (FGD2)

### Internal opportunities of a CoP

Participants identified several opportunities that enhance CoP functioning which could be grouped into three overarching areas. The first group centres on knowledge diversity and shared expertise. Participants reported that CoPs benefit from the multidisciplinary backgrounds of members, their geographic diversity, and international connections that facilitate cross-learning. The CoPs serve as repositories of collective knowledge, enabling continuous sharing and mutual learning. As one participant explained:

“*Also, it fosters collaborative learning, but also the CoPs, they connect diverse stakeholders including healthcare providers, policymakers, NGOs, foster collaboration that strengthen advocacy*.” (P02)

At the interpersonal level, participants emphasised the value of safe spaces for open dialogue, the passion and shared commitment among members, and the emotional and professional support embedded in their social networks:

“*Which really fosters a collective knowledge and also the open communication that is there. In Share-Net we encourage transparent and regular communication because it fosters trust and collaboration among members*.” (P02)

Finally, participants identified opportunities related to strategic positioning, visibility, and influence. These included the ability to avoid duplication of work through improved coordination, the autonomy members have to take the initiative, the influence of powerful voices within the network, and the growing visibility and uniqueness of each CoP. Additionally, participants noted the benefits of Share-Net’s centrally provided resources, which strengthened collaboration, communication, and learning across CoPs:

“*What we have seen in Share-Net is that we have different stakeholders. But at some point we notice that some of them are working on the same interventions in the same area in the same field. And they don't know each other. So when we bring them together, they know, oh, this person is doing this. So we should do something together so that we avoid duplication of intervention 'cause we are targeting the same people, we are going to the same area. So it's just to really bring them together and do something as one, instead of duplicating things over and over*.” (P02)

During the FGD, all seven participants agreed that CoPs are effective for KT, highlighting opportunities that are also detailed in Supplementary file 3. However, some noted the need for improvement.

### Internal challenges of a CoP

There were a number of challenges identified by participants which can be grouped into three overarching issues. The first group of challenges relates to structural and operational factors. Participants described limitations in the coordination and facilitation of CoP activities, including difficulties with allocating roles and responsibilities, ensuring follow-up after meetings, and maintaining continuity in communication and planning. This often affected the consistency of CoP meetings and the timely organisation and implementation of activities. Participants also highlighted insufficient monitoring and evaluation structures, as well as constrained financial and human resources, which limited the CoPs’ ability to sustain activities over time. In addition, several participants described a lack of clear goal-setting and strategic direction within some CoPs, leading to uncertainty among members about the CoP’s priorities, intended outcomes, and what could realistically be achieved collectively. One participant commented:

“*I would say finance is a challenge because if the CoP objective is not only learning and exchanging, but sometimes, for example in our CoP, we had some big ambitions to conduct a research, but then we realised that the small budget we had from Share-Net was too small to cover the cost of this study*.” (P07)

It became evident that even among CoPs with clear goals and ambitions, limited budgets often hindered the realisation of these objectives. However, financial constraints were only one aspect of the broader structural and operational challenges identified by participants. Participants shared that other kinds of capacity issues also impacted their ability to do work within the CoP and that these capacity issues were not only financial, but also “* … capacity in terms of human resources, in terms of finances, in terms of logistics*.*”* (P13)

In order for CoPs to effectively carry out their plans and activities, they needed more human resources support from the Share-Net secretariats and from other CoP members themselves, as well as logistical support in bringing the CoP activities to fruition.

Participants also noted challenges related to limited institutional memory, caused by frequent turnover among members and, at times, within Share-Net secretariat staff, which affected continuity and strategic planning. One participant further reflected on the sheer scale and complexity of the issues CoPs aim to address as an additional challenge.

A second set of challenges reflects strategic positioning and influence. Many CoPs struggled to establish their reputation within the SRHR field, while also navigating competition between organisations within the CoP. Participants described limited power or authority to influence policy processes, all of which constrained the wider reach and impact of their work. As Share-Net primarily functions as a knowledge network, its focus lies on knowledge management and exchange rather than direct advocacy. Consequently, policy influencing was often left to organisations whose core work involves lobbying and advocacy. The extent to which a CoP could engage in policy-related activities therefore depended largely on the capacity, expertise, and networks of its members. Many CoP members were individuals, researchers, or representatives of community-based organisations and smaller NGOs who were interested in influencing policy, but who did not necessarily have established relationships, resources, or access points to engage policymakers directly:

“*In general, I would say there's a lot of organisations that are very interested in translating knowledge into policy and practice, but it's very, very hard. We work a lot with community based organisations, they see this as a dream, to see something translated in policies*.” (P03)

The third theme concerns relational and participation dynamics. CoPs often had to manage diverse and at times conflicting member priorities, sustain engagement in a voluntary structure, and overcome reduced participation over time. One participant acknowledged that “*It's very, very hard to keep members engaged and working through the whole year*” (P03) and continued to explain that CoPs need to think of ways to keep members active:

“*I think the problem is, as I said before, the main issue to translate knowledge is seeing the CoP as it is, so we need to see how to keep members engaged? And this means probably giving them a different role in the CoPs or to seek different ways of working with them*.” (P03)

Some CoPs also reflected on the risk of operating within a relatively “safe bubble,” where strong internal cohesion and shared perspectives could unintentionally limit engagement with broader audiences and diverse contextual realities. A participant noted that ideas and materials developed within CoPs were not always equally accessible, relevant, or feasible in contexts where SRHR priorities, sociocultural norms, or political conditions differed substantially.

### Contextual factors that affect the CoPs functioning

Numerous contextual factors were identified to affect the CoPs functioning. Supplementary File 4 presents the contextual factors influencing KT processes, including the corresponding codes, themes, and descriptions. Power, legal and political dynamics were identified as a key challenge in the KT process, with many emphasising how broader institutional structures determine what knowledge is legitimised, shared, or suppressed. Several interviewees reflected on how colonial legacies continue to influence global SRHR work through unequal North–South power relations, donor-driven agendas, and perceptions of SRHR as a “Western” concept imposed on other contexts. Participants also described how patriarchal norms shaped KT processes, for example through male-dominated decision-making structures, restrictive gender norms, and the marginalisation of women’s and gender-diverse perspectives in SRHR discussions and implementation. Political resistance and restrictive legal environments formed a related set of challenges, with participants describing how changing political climates, conservative movements, restrictive laws, political instability, and violence constrained KT processes.

During one FGD, participants discussed how patriarchal systems, colonial legacies, funding inequalities, and government influence shaped the translation and uptake of SRHR knowledge. Reflecting on these interconnected dynamics, one participant stated:

“*Power imbalances or power dynamics are kind of ever present. I would say like an ever present factor in everything that is on the side and everything we do. I just, I feel like the imbalances are like unequal power dynamics obviously is like an overarching issue that that kind of permeates through most of these points*.” (FGD 2)

In addition, crises and broader societal disruptions were reported to further destabilise KT processes. Climate change, the COVID-19 pandemic, economic volatility, and periods of political fragility all diverted attention and resources away from KT activities and disrupted continuity.

Safety concerns also prevented some individuals from engaging openly in SRHR work, particularly in restrictive settings, with one member explaining:

“*It’s unsafe. I do believe all the CoP members, they have the same values, they believe in human rights. But the reality is even if you believe in human rights, you don't want to expose yourself as an organisation or as an individual. For example myself, I do believe in human rights, in people having the right to choose. But for example, if you want me to go and talk about LGBTQI +  in Senegal, for my safety that is something I would not do*.” (P07)

Participants also described how these dynamics intersected with sociocultural norms and belief systems, such as conservatism, religious opposition, and deeply rooted taboos, which made it difficult to address sensitive SRHR topics or reach more conservative communities: “*Beliefs, religion, opinions have an influence on the implementation of SRHR projects*.” (P10)

Participants also pointed to structural and resource constraints, including the challenges of working in rural areas where there can be limited infrastructure, lower literacy levels among communities they are working with, as well as language barriers in cases where countries have multiple local languages, all of which reduced these communities’ access to information and hindered KT.

Bureaucratic procedures and shifting governmental priorities further constrained KT efforts, particularly when governments deprioritised SRHR-related activities in favour of other political agendas. Participants described how changes in government priorities could delay the approval of knowledge products, reduce funding opportunities, limit stakeholder engagement, and affect which SRHR topics were considered politically acceptable or relevant.

Digital censorship and online restrictions were likewise described as barriers that undermined dissemination and limited the reach of knowledge products. When discussing a website designed for sexuality educators, one participant explained the impact of digital censorship on its dissemination:

“*It's more about what are the guidelines, what the criteria are for the platform itself. So we obviously see Instagram faces a lot more censorship than X … but I think what we notice from country to country is that a country's internet regulatory laws very much played a role in how our website was blocked*.” (P06)

Despite these obstacles, participants highlighted several contextual factors that facilitated progress. Societal acceptance, activism, and progressive social movements were described as important enablers that opened dialogue and created momentum for change:

“*But we have experienced activism just exploding out of nowhere for every reason. These strong activism societies can be very helpful because people feel empowered to do stuff*.” (P03)

Supportive government policies, such as national guidelines for family planning services and comprehensive sexuality education, favourable legal frameworks, and other catalytic events, such as the decriminalisation of abortion up to 24 weeks in Colombia in 2022, for example, also created windows of opportunity for more effective KT and implementation.

### Strategies to address the challenges

The recommendations to the challenges identified during the interviews and further explored and prioritised during the FGDs could be grouped into six broader categories, related to: dissemination, M&E, member engagement, resources, adaptation to context and other themes. Supplementary File 5 presents strategies to address identified challenges, including the corresponding codes and descriptions.

To address challenges related to the dissemination of CoP knowledge products, participants emphasised the importance of developing structured dissemination strategies and ensuring that knowledge products were designed in accessible and inclusive formats appropriate for diverse audiences. Strengthening internal dissemination could foster cross-learning within Share-Net, while external efforts could leverage platforms like knowledge fairs or local media to broaden outreach:

“*To address the challenge of dissemination and not reaching the target population, so I think accessibility is a consideration, it can be one of the strategies because by ensuring that the materials are accessible to all you know, including those with a disability, a language barrier or low literacy levels. So this might involve translating materials or using local languages. By doing this, we can overcome this challenge*.” (FGD 2)

Developing a CoP M&E system was identified as another critical priority. Participants recommended establishing baseline measures before initiating CoP activities, such as assessing existing beliefs, attitudes, and levels of community support around specific SRHR topics, for example support for comprehensive sexuality education. This was considered important for tracking changes over time and evaluating the impact of specific CoP activities more effectively, and also to monitor specific activities of the CoP. This was highlighted by a participant who shared “*I think that's something we need to start doing first, learning how to do monitoring and evaluation. Start including these components in the design of the products of the CoPs*” (FGD 1). They also emphasised aligning future M&E frameworks with global benchmarks like the SDGs and incorporating feedback mechanisms. Suggestions included dedicated funding for M&E within projects and forming a specialised CoP on M&E.

To boost member engagement, participants suggested promoting membership through targeted (internal) advertising, highlighting tangible benefits of participation, and fostering appreciation and respect among members. One participant shared how this was done:

“*For us it has worked a lot to provide a very specific package of benefits for members because sometimes, and especially when we want to people working in CoPS, it's not very clear [what] they as a volunteer are getting from working in a CoP. It's more work. So of course we need to be very … For example, we ensure we provide free training courses or workshops or events that are exclusive for members*.” (FGD 1)

The value of in-person meetings was highlighted for building deeper connections, while maintaining ongoing engagement through regular updates and events ensures sustained participation. Encouraging member-driven motivation and improving KT processes were also seen as essential to retaining active involvement.

To address resource challenges, co-creation and partnerships were identified as ways to optimise resource use by pooling expertise, sharing costs, and leveraging existing networks and infrastructure. Participants also recommended a fundraising strategy that would secure diverse funding from local sources, private-sector partnerships, or even paid services. This was highlighted by one participant who shared:

“*If you are facing a challenge regarding resources, we can partner with other organisations or individuals to share resources and skills. Collaborative efforts can maximize impact without requiring additional resources*.” (FGD 2)

Participants stressed that resource constraints should not hinder CoP activities, emphasising efficient resource management.

Participants emphasised that contextual factors play a critical role in shaping KT strategies and stressed the need to adapt activities to the sociopolitical and cultural context. Examples included restrictive legal and political environments, cultural and religious norms surrounding SRHR, and language differences. Adaptation involved tailoring messaging to local norms and policy priorities, engaging stakeholders early to ensure relevance and buy-in, and adjusting the format and timing of activities according to available resources and political feasibility.

Cross-learning within Share-Net and with external organisations was described as a key mechanism to support this adaptation, enabling CoPs to draw on experiences from other contexts, reflect on what strategies were effective or ineffective, and modify approaches accordingly. Participants highlighted sustaining long-term initiatives and crafting strategic messaging as important. In addition, participants identified broader strategies to address contextual challenges, including advocacy to respond to policy gaps, linking CoP activities to national and global priorities, and strengthening Share-Net’s facilitation role to better support coordination and adaptation across contexts.

## Discussion

This study explored how Share-Net's CoPs in various countries facilitate the translation of knowledge into policy and practice within the field of SRHR, and identified the contextual factors influencing these efforts. Four key findings emerged: (1) CoPs focus strongly on creating knowledge products while structured dissemination, monitoring and evaluation (M&E) remained limited; (2) CoPs face internal structural and interpersonal challenges together with important opportunities; (3) contextual factors, especially power dynamics, political environments, and sociocultural norms, strongly influence KT processes; and (4) participants proposed practical strategies to strengthen CoP functioning and KT.

A key insight from this study is that Share-Net’s CoPs primarily focus on the creation of knowledge products. While this represents an important step in the KtA framework, our findings suggest that knowledge production alone does not translate into effective, meaningful changes in policy and practice. Despite covering a wide range of SRHR topics, most participants did not report topic-specific policy or programmatic outcomes, limiting insight into differentiated CoP impact. While similar patterns have been observed in other KT and CoP studies,^30,31^ our findings suggest that this reflects a structural tendency within CoPs to prioritise development of knowledge products over knowledge use. This is not only a question of effectiveness in translating evidence into policy and practice, but also of power, as the uptake of knowledge depends on which actors, forms of expertise, and priorities are recognised and valued by policymakers, funders, and institutions involved in SRHR decision-making.

In our study, the absence of robust M&E frameworks further complicates efforts to assess whether outputs (i.e. knowledge products) have led to meaningful short- to medium-term outcomes (i.e. changes in behaviours of specific stakeholders) or contributed to long-term impacts (i.e. sustained change). Rather than solely aiming for and measuring high-level systemic transformation, our findings indicate that CoP impact may be more diffuse and incremental, operating through individual and relational pathways such as strengthened skills, professional networks, and collaborative capacities. These forms of capacity strengthening are difficult to measure or directly attribute, yet they may represent key mechanisms through which CoPs influence practice and policy over time. This highlights the need to move beyond linear models of KT toward learning-oriented Monitoring, Evaluation and Learning (MEL) approaches that capture iterative processes and relational change. Such approaches align with the KtA framework's emphasis on continuous learning cycles, where dissemination is embedded within ongoing adaptation rather than treated as a standalone activity.^27^ Embedding learning-oriented MEL within CoPs could enhance their ability not only to generate knowledge, but also to adapt, reflect, and demonstrate their contributions to broader SRHR goals. For example, CoPs could combine routine documentation of activities and outputs, such as workshops, campaigns, training sessions, or dissemination of knowledge products, with ongoing reflection and feedback from members, communities, and stakeholders on how these activities are received, used, and adapted in practice. Monitoring engagement, participation, and uptake over time could help CoPs identify which approaches are effective, for whom, and under which contextual conditions, enabling them to continuously refine their KT strategies. In this way, MEL becomes not merely a reporting exercise, but a continuous learning process that strengthens the responsiveness, relevance, and long-term impact of CoP activities. It may also support CoPs and implementation researchers in better understanding and communicating the mechanisms through which CoPs contribute to KT as part of routine practice, while reducing reliance on external resources, particularly in contexts where KT remains under-supported.^26,32,33^

A second key finding is that CoPs experience a combination of internal structural and interpersonal challenges, including limited facilitation, unclear goals, fluctuating member engagement, and resource constraints. The recurrence of these challenges across CoPs suggests that they are not isolated operational issues, but underlying organisational conditions that may limit the continuity, coordination, and long-term sustainability of collaborative KT efforts. Similar internal barriers have been documented in research on CoPs in health networks, particularly in voluntary or under-resourced settings.^18–20,33,34^ Therefore these need to be anticipated at the design and implementation stages of CoPs.

Participants identified important opportunities offered by CoPs such as multidisciplinary expertise, safe spaces for dialogue, shared purpose, international links, and reduced duplication of efforts. The CoPs were characterised by dynamic and adaptable priorities, voluntary participation, and limited formal structures or predefined roles. Participants described how this openness enabled individuals from different disciplines, sectors, and contexts to engage in ways that matched their expertise, needs, and availability, thereby supporting inclusivity. At the same time, the absence of clear structures sometimes undermined continuity, coordination, and the ability to demonstrate measurable impact. These strengths echo previous studies showing that CoPs can foster collaborative learning, mutual support, and innovation.^18,19,35,36^ This tension reflects what participants described as a “beautiful chaos”: a fluid and adaptive structure that enables inclusivity and responsiveness, but can also lead to instability and limited accountability. Strengthening facilitation, clarifying goals, and securing resources may therefore be critical to translating the collaborative potential of CoPs into more sustained and effective KT outcomes.

The influence of contextual factors emerged as a third key finding, highlighting that KT within CoPs is deeply shaped by broader power structures, operating at local, national, and global levels, including political, cultural, religious, social, gendered, economic, and historical systems and norms. Participants described how patriarchal norms that restricted open discussion of SRHR, religious structures that shaped the acceptability of SRHR topics, restrictive political and legal environments that limited advocacy and dissemination, colonial legacies that influenced power relations and whose knowledge was valued, and resource constraints such as limited funding and infrastructure affected not only the feasibility of KT activities, but also whose knowledge was legitimised, shared, and acted upon. These findings align with broader KT and global health literature showing that KT processes are shaped by sociopolitical and structural power dynamics, including institutional hierarchies, resource inequities, and epistemic injustices, which influence whose knowledge is legitimised, shared, and acted upon,^37–40^ underscoring that KT is not a neutral or purely technical process. From a rights-based perspective, these power dynamics reflect deeper inequities in participation, representation, and access to information, which may result in the systematic exclusion of certain groups from knowledge production and decision-making processes, i.e. risk of an epistemic injustice.^41,42^ This suggests that CoPs operate within, and are shaped by, existing power structures that fundamentally influence their ability to contribute to equitable SRHR outcomes.

Despite these constraints, our findings illustrate that supportive policies, activism, and shifts in social norms can create openings for KT within otherwise constrained environments. Similar dynamics have been observed in other studies, which demonstrate that enabling political and social conditions can accelerate KT and strengthen progress in SRHR.^43–45^ These findings highlight that any effort to strengthen KT in CoPs must account for the broader sociopolitical and cultural conditions that fundamentally shape whose knowledge can be mobilised and applied.

The final key finding is that participants identified practical, context-sensitive strategies to strengthen KT within CoPs. These included structured dissemination planning, learning-oriented M&E systems, diversified resource mobilisation, enhanced facilitation, in-person engagement, and stronger adaptation to local contexts. Comparable recommendations appear in KT and implementation science literature, which emphasise iterative learning, adequate resourcing, and participatory engagement as central to successful KT.^39, 46^ Existing studies similarly highlight that effective KT requires attention not only to tools and processes, but also to the capabilities of those leading and implementing KT activities.^18,^^47–49^ Importantly, participants’ proposed strategies reflect bottom-up, practice-driven solutions grounded in experiential knowledge, rather than externally imposed models. This suggests that CoP members possess a nuanced understanding of how to strengthen KT, but are constrained by systemic and organisational limitations, such as limited resources, insufficient institutional support, competing priorities, and power dynamics. Supporting such locally grounded approaches may therefore be key to enhancing the relevance, effectiveness, and sustainability of CoPs in diverse SRHR contexts. Collectively, these findings underscore the importance of context-sensitive, well-resourced, and equity- and rights-oriented approaches that actively address power imbalances and support meaningful inclusion in KT processes.

Taken together, this study contributes to existing literature by offering a more nuanced understanding of how CoPs function as KT mechanisms in SRHR. Rather than acting as direct drivers of policy change, our findings suggest that CoPs operate as relational and capacity-building platforms, where impact occurs incrementally through strengthened networks, skills, and collaboration. These insights extend existing KT and CoP literature by shifting the focus from outcome attribution towards understanding the relational and contextual mechanisms through which change occurs. In addition, this study highlights that KT within CoPs is not a neutral or purely technical process, but is deeply shaped by power dynamics and sociopolitical contexts that influence whose knowledge is prioritised and applied. The identification of CoPs as a form of “beautiful chaos” further contributes a conceptual lens to understand the inherent tension between flexibility and structure, where adaptability may support inclusivity but also limit consistency and measurable impact. Finally, the study foregrounds practice-driven, context-sensitive strategies proposed by CoP members themselves, underscoring the importance of bottom-up approaches to strengthening KT in diverse SRHR settings.

### Limitations

While this study provides valuable insights, some limitations must be considered. The research focused on a single network, which may restrict the generalisability of findings to other CoP models or SRHR networks. Another limitation is the snowball sampling, which might have introduced selection bias, favouring active or positively inclined participants while excluding less active or critical voices. The reliance on English proficiency excluded perspectives from non-English-speaking members, such as those in Burkina Faso, as no reliable translation resources were available. Conducting online discussions via Microsoft Teams introduced challenges, including unstable internet access and limited ability to observe non-verbal cues, particularly when cameras were off. The FGD included only seven of the fifteen participants, which may have restricted the discussion of certain strategies or viewpoints. Although both staff and members were represented, the sample largely consisted of highly engaged participants, which may have influenced the perspectives shared in this study. Additionally, data analysis was conducted by a single researcher with limited cross-checking, which could impact the reliability of theme identification despite regular team discussions. Ethical considerations also necessitated the exclusion of sensitive data that participants felt unsafe discussing in the context of their CoP work.

Another limitation stems from Share-Net’s position as the host organisation of the CoPs examined. Such institutional proximity could have shaped participant responses, potentially leading to more favourable portrayals of CoP functioning or reduced willingness to express criticism. While mitigation strategies, including the use of an independent lead researcher, assurances of confidentiality, and member-checking, were employed, the possibility of residual influence from the funding organisation remains.

Furthermore, the transdisciplinary approach also presents several challenges. It carries the risk of potential biases, such as the prioritisation of organisational perspectives or interests. To mitigate this, measures included appointing an independent lead researcher, fostering academic partnerships, and holding regular team discussions to ensure critical reflection.

Lastly, the loss of Share-Net’s primary funding during the study may have influenced CoP activities and participant perspectives, adding uncertainty to findings related to resource availability.

### Strengths

This study benefited from several key strengths that contributed to its robustness and the richness of its findings. Our transdisciplinary approach, in which CoP members and facilitators were also co-researchers, specifically in the member-checking process, ensured that the data were accurate and that trust was strengthened among participants. While their involvement did not extend to the broader research process, they did contribute to the development of this article by providing input and reviewing sections of the manuscript, which helped enhance its clarity and relevance. The use of snowball sampling and diverse participant backgrounds allowed for a broad range of perspectives, enhancing inclusivity. Additionally, the study’s geographical diversity captured insights from various global contexts, enriching the findings with a wide variety of experiences. Finally, the study also benefited from Share-Net's extensive and long-standing networks, which provided valuable real-world complexity and insights drawn from lived experiences. Credibility was further enhanced through triangulation across data sources, researcher perspectives, and member-checking procedures.

## Conclusion

Overall, this study highlights that the value of CoPs lies not only in knowledge production, but in their potential to support relational and context-responsive pathways to change. Strengthening CoPs therefore requires moving beyond linear models of KT toward approaches that explicitly address power, support meaningful inclusion, and align with the broader goal of advancing equitable SRHR outcomes.

### Recommendations for future efforts

Participants proposed several practical strategies to strengthen CoPs, including improved dissemination planning, learning-oriented M&E, enhanced facilitation, diversified resource mobilisation, stronger member engagement, and adaptation to local contexts. Building on these findings, we suggest that future efforts should also address the broader governance and power-related challenges shaping KT processes. Strengthening CoPs may require balancing contextual responsiveness with clearer coordination, continuity, and accountability. Rather than relying solely on voluntary participation, CoPs may benefit from more structured facilitation that supports sustained engagement and follow-up. Participants also emphasised the importance of cross-learning, stakeholder involvement, strategic messaging, and clear member benefits. Examples included organising Knowledge Fairs and exchange activities between Share-Net networks, engaging policymakers and community stakeholders early in CoP activities, tailoring messages to different target audiences, and offering tangible benefits such as training, networking opportunities, and access to small grants. These strategies suggest that sustained engagement depends not only on individual motivation, but also on whether members perceive CoPs as meaningful spaces for collaboration and exchange.

Beyond internal governance, CoPs must also navigate the power structures within which KT takes place. Strengthening connections between community-based knowledge, implementation practice, and policy-making processes may improve the relevance and uptake of KT activities. This could involve engaging policymakers, civil society actors, and affected communities earlier and more consistently, while adapting dissemination strategies and knowledge products to local contexts. Such approaches may help ensure that locally grounded and underrepresented perspectives are better reflected in SRHR policy and practice.

Embedding learning-oriented MEL approaches within routine CoP activities may further support continuous reflection, adaptation, and collective learning. Rather than functioning primarily as reporting tools, MEL processes could help CoPs understand how knowledge products and engagement strategies are received, adapted, and applied across contexts, and identify which approaches are effective, for whom, and under which conditions.

Future research should prioritise longitudinal approaches that examine how CoP activities influence relationships, policy engagement, implementation processes, and practice over time. Such research could support the development of MEL frameworks that better capture the iterative, relational, and context-dependent nature of KT within SRHR.

## Supplementary Material

Supplemental Material

## Provenance statement

This article was not commissioned and went through external peer review.
