# Supplementary material for: “A beautiful chaos”: a qualitative study on exploring the role of international communities of practice in knowledge translation for sexual and reproductive health and rights
Source: Sex Reprod Health Matters. 2026 Aug 17;33(1):2714691. doi: 10.1080/26410397.2026.2714691 (PMC13700466; doi:10.1080/26410397.2026.2714691)
Supplement: Supplementary file 1 [file ZRHM_A_2714691_SM8139.docx]

***Supplement 1: Interview scripts***

Interview script CoP members

| Objective | Time schedule | Question | Objective |
| --- | --- | --- | --- |
| Preparation | Prior to FGD | Send out a reminder email to participants.  Ensure the participants are aware of the websites that will be used and that they have access | Ensure that everything is adequately prepared prior to the interview |
| Introduction | 5 min | **Introduction**  "Hello [name participant], and thank you for joining this interview today. I really appreciate you taking the time out of your schedule to be here.  To introduce myself briefly, my name is Noa, and I am conducting this research as part of my Master’s Medicine Research Internship. Before we begin, I’d like to give you an overview of today’s session. As mentioned in the email, this research aims to explore how Communities of Practice (CoPs) facilitate knowledge translation and what contextual factors influence this process.  The interview will last approximately 60 minutes, during which I’ll ask you about your experiences with your CoP, knowledge translation, and the contextual factors that shape these processes. When I refer to 'knowledge translation' in this context, I mean translating knowledge into policy and practice. Another term linked to this concept is knowledge dissemination.  Does that sound clear so far?  Please remember that your participation today is completely voluntary. If there’s any question or activity you’d prefer not to engage in, just let me know, and that will be fine. You’re also free to withdraw at any point, either during or after the research is completed. If you have any questions or concerns at any time, don’t hesitate to ask.  Before we proceed, I’d like to request your permission to record this session. The recording will help me keep track of everything discussed, and any transcriptions will remain anonymous, with no personal information identifying you. Do I have your consent to record the interview?  Any questions before we get started?"  **Action** *Click record!* | Welcoming participant, clarifying  the aim of the session, the research  context,  how the session will be conducted and informed consent |
| Introduction  participant | 5 min | **Introduction**  So to start off the interview, I first wanted to ask you if you could please introduce yourself, your CoP, and briefly describe your role in the CoP? | General background and expertise |
| CoP | 5 min | **Introduction**  Okay, thank you for your answer, that sounds cool!  To go a little bit more in depth about your CoP, I was wondering how your CoP is structured and organized?  **Potential follow-up questions**   - What is the goal of your CoP? - How would you describe the team(work) within your CoP? - How does your CoP get to work? - Who are the key stakeholders that your CoP engages with, both within and outside the group? - How does your CoP get funding? | Explore how the CoP is structured and organized. |
|  |  |  |  |
| Knowledge translation (x) | 15 min | **Introduction**  Thank you for your answer, everything is clear for me! Now I want to move on to the next topic, ‘knowledge translation’. So to start off with a very broad and general question.  How does your CoP facilitate the translation of knowledge into dissemination, policy and practice within the SRHR field?  **Potential follow-up questions**  1. How did you identify the main problems or challenges your Community of Practice (CoP) should focus on?  2. What process did you use to find, select, and review the relevant knowledge or research for your CoP's topic?  3. What knowledge translation activities did you identify for these in policy, practice or dissemination?  4. Did you find available knowledge sufficiently suitable for your context to use without adaptation? If not, how did you adapt the available knowledge to fit your local context?  5. Can you describe your approach to implementing the knowledge or interventions within your CoP?  6. Are you monitoring and evaluating the outcomes of the implementation? If so, how? Which were these outcomes?  **ACTION:** try to summarize their input | Explore how the CoP translates knowledge via the action-to-knowledge model. |
| Barrières | 5 min | **Introduction**  I am also curious, what challenges does your CoP face in translating knowledge into policy and practice?  **Potential follow-up questions**  Can you name an example?  Are there any specific areas related to your Community of Practice (CoP) where you encountered knowledge gaps, i.e. more knowledge or information was required than available? If so, how do you deal with these? | Explore the potential barriers |
| Opportunities | 5 min | **Introduction**  I was also wondering the other way around: What factors help facilitate knowledge translation within your CoP?  **Potential follow-up questions**  Are there specific strategies, resources, or collaborations that have been particularly effective? | Explore the opportunities. |
| Sustain and improvement | 5 | **Introduction**  So we already talked about barriers and opportunities. I was also wondering what strategies do you think are necessary to sustain and improve knowledge translation efforts within your CoP?  **Potential follow-up questions**  Can you name an example? | Explore which strategies sustain and improve knowledge translation. |
| Contextual factors (x) | 10 | **Introduction**  Now I want to move on to the last topic, contextual factors. Do you know what factors within your country or local context influence the effectiveness of knowledge translation in SRHR?  **Potential follow-up questions**  Think about factors such as:   - Structural (Physical Infrastructure, information technology infrastructure, work infrastructure) - Relational connections - Communications - Culture (human equality centeredness, recipient centeredness, deliverer centeredness, learning centeredness) - Tension for change - Compatibility - Relative priority - Incentive systems - Mission alignment - Available resources (funding, space, materials & equipment) - Access to knowledge and information   Can you name an example?  Did you have any interactions with other countries? If so, based on your interactions with CoPs in other countries, how do knowledge translation efforts differ across contexts? | Explore the contextual factors that influence knowledge translation. |
|  |  |  |  |
| Added comments | 3 min | **Introduction**  So we are almost at the end of the interview. My last question is based on your experiences, what would be a key recommendation you would give others about knowledge translation activities?  **Potential follow-up questions**  Is there anything else you would like to add about the role of CoPs in knowledge translation within SRHR? | Get one key recommendation and check if the participant has anything else to add to the interview. |
| Ending | 2 min | **Introduction**  We have come to the end of the interview. I wanted to thank you for your valuable input. I will send you a summary of the main findings as soon as possible as a member check via email.  And I hope to see you at the focus group discussion! If you have any questions in the meantime, please feel free to contact me!  Have a good day! | Round up the interview and explain the next steps. |

*Interview script facilitator*

| Objective | Time schedule | Question | Objective |
| --- | --- | --- | --- |
| Preparation | Prior to FGD | Send out a reminder email to participants.  Ensure the participants are aware of the websites that will be used and that they have access | Ensure that everything is adequately prepared prior to the interview |
| Introduction | 5 min | **Introduction**  "Hello [name participant], and thank you for joining this interview today. I really appreciate you taking the time out of your busy schedule to be here.  To introduce myself briefly, my name is Noa, and I am conducting this research as part of my Master’s Medicine Research Internship. Before we begin, I’d like to give you an overview of today’s session. As mentioned in the email, this research aims to explore how Communities of Practice (CoPs) facilitate knowledge translation and what contextual factors influence this process.  The interview will last approximately 60 minutes, during which I’ll ask you about your experiences with your CoPs, knowledge translation, and the contextual factors that shape these processes. When I refer to 'knowledge translation' in this context, I mean translating knowledge into dissemination, policy and practice. I will also take some notes during this interview, so sometimes you see me looking down, but then I am writing.  Does that sound clear so far?  Please remember that your participation today is completely voluntary. If there’s any question or activity you’d prefer not to engage in, just let me know, and that will be fine. You’re also free to withdraw at any point, either during or after the research is completed. If you have any questions or concerns at any time, don’t hesitate to ask.  Before we proceed, I’d like to request your permission to record this session. The recording will help me keep track of everything discussed, and any transcriptions will remain anonymous, with no personal information identifying you. Do I have your consent to record the interview?  Any questions before we get started?"  **Action** *Click record!* | Welcoming participant, clarifying  the aim of the session, the research  context,  how the session will be conducted and informed consent |
| Introduction  participant | 5 min | **Introduction**  So to start off the interview, I first wanted to ask you if you could please introduce yourself, describe your role as facilitator and your countries CoP’s that you are responsible for? | General background and expertise |
| CoP | 5 min | **Introduction**  Okay, thank you for your answer, that sounds cool!  To go a little bit more in depth about the CoP’s, I was wondering how your country's CoPs are structured and organized?  **Potential follow-up questions**   - What are the goals of your CoP? - How would you describe the team(work) within the CoPs? - How do the CoPs get to work? - Who are the key stakeholders that the CoPs engage with, both within and outside the group? - How does the CoPs get funding? | Explore how the CoP is structured and organized. |
|  |  |  |  |
| Knowledge translation (x) | 15 min | **Introduction**  Thank you for your answer, everything is clear for me! Now I want to move on to the next topic, ‘knowledge translation’. So to start off with a very broad and general question.  How do the CoPs of your country facilitate the translation of knowledge into dissemination, policy and practice within the SRHR field?  **Potential follow-up questions**  1. How did you or your members identify the main problems or challenges a Community of Practice (CoP) should focus on?  2. What knowledge translation activities did you/they identify for these in policy or practice?  3. What process did you/they use to find, select, and review the relevant knowledge or research for your CoP's topic?  4. Did you/they find available knowledge sufficiently suitable for your context to use without adaptation? If not, how did you adapt the available knowledge to fit your local context?  5. Can you describe your/their approach to implementing the knowledge or interventions within your CoP?  6. Are you/they monitoring and evaluating the outcomes of the implementation? If so, how? Which were these outcomes?  **ACTION:** try to summarize their input | Explore how the CoP translates knowledge via the action-to-knowledge model. |
| Barrières | 5 min | **Introduction**  I am also curious what challenges do the CoPs of your country face in translating knowledge into policy and practice?  **Potential follow-up questions**  Can you name an example?  Are there any specific areas related to your Community of Practice (CoP) where you encountered knowledge gaps, i.e. more knowledge or information was required than available? If so, how do you deal with these? | Explore the potential barriers |
| Opportunities | 5 min | **Introduction**  I was also wondering the other way around: What factors help facilitate knowledge translation within the CoPs of your country?  **Potential follow-up questions**  Are there specific strategies, resources, or collaborations that have been particularly effective? | Explore the opportunities. |
| Contextual factors (x) | 10 | **Introduction**  Now I want to move on to the last topic, contextual factors. Do you know what factors within your country or local context influence the effectiveness of knowledge translation in SRHR?  **Potential follow-up questions**  Think about factors such as:   - Structural (Physical Infrastructure, information technology infrastructure, work infrastructure) - Relational connections - Communications - Culture (human equality centeredness, recipient centeredness, deliverer centeredness, learning centeredness) - Tension for change - Compatibility - Relative priority - Incentive systems - Mission alignment - Available resources (funding, space, materials & equipment) - Access to knowledge and information   Can you name an example?  Did you have any interactions with other countries? If so, based on your interactions with CoPs in other countries, how do knowledge translation efforts differ across contexts? | Explore the contextual factors that influence knowledge translation. |
| Sustain and improvement | 5 | **Introduction**  So we already talked about barriers and opportunities, I was also wondering what strategies do you think are necessary to sustain and improve knowledge translation efforts within the CoPs of your country?  **Potential follow-up questions**  Can you name an example? | Explore which strategies sustain and improve knowledge translation. |
| Added comments | 3 min | **Introduction**  So we are almost at the end of the interview. My last question is based on your experiences, what would be a key recommendation you would give others about knowledge translation activities?  **Potential follow-up questions**  Is there anything else you would like to add about the role of CoPs in knowledge translation within SRHR? | Get one key recommendation and check if the participant has anything else to add to the interview. |
| Ending | 2 min | **Introduction**  So we have come to the end of the interview. I wanted to thank you from the bottom of my heart for your team and your valuable input. I will send you as soon as possible a summary of the main findings as a member check via email.  And I hope to see you at the focus group discussion! If you have any questions in the meantime, please feel free to contact me!  Have a good day! | Round up the interview and explain the next steps. |

***Supplement 2: Focus Group Discussion script***

| Objective | Time schedule | Question | Objective |
| --- | --- | --- | --- |
| Preparation | Prior to FGD | Send out a reminder email to participants.  Ensure the participants are aware of the websites (Miro & Teams) that will be used and that they have access | Ensure that everything is adequately prepared prior to the FGD. |
| Introduction | 15:00-15:10 | **ACTION:**  Most likely, participants will enter the online environment at different times.  They are welcomed and we have small talk until everybody has arrived/ it is time to start the FGD  **INTRODUCTION**  **¨**Hello everyone. First of all, I would like to welcome you all to this focus group discussion. I am really happy that you were all able to make it today. I understand you all have busy schedules, so thank you for taking the time to be here.  As you are all aware, my name is Noa and I have been working on this project as a part of my Master Research Internship. I am going to start off by telling you a bit more about the aim and programme of today. As you all have been an interviewee, you are all aware that this research is being conducted to gain a better understanding in how Community of Practices facilitate knowledge translation and which contextual factors influence these. Through these focus groups, I hope that we will be able to think and work together towards this goal. We have a lot of people with a wide range of expertise and background, so I am hoping these sessions will allow you all a great opportunity to learn from each other and discuss this topic. Today we will discuss the preliminary results and have some brainstorm sessions about the challenges and contextual factors.  If you want to talk, please raise your hand in Teams, so that I can see who wants to talk. That way I can moderate the discussion best to my abilities.  Does anybody have any questions?  Then also a small reminder that this session will be recorded, Let me know if anybody obtains from this?  Click on record  Then I also want to introduce Julia, she is an amazing researcher in the Netherlands and she will be helping moderate this session. So if you have any technical difficulties you can ask her.  Then I want to do a quick round of introduction. If everybody could briefly state their name, background, country and CoP?  So the program we will be using today is Mural. You can move around the program with your mouse. On the left side of the screen is a black box with icons. The second icon is the most important. If you click on there, you can get sticky notes. A sticky note appears on the screen and you can change the colour, size and also write in it. Each participant has assigned their own colour. You can also see the instructions in slide 0.4. |  |
| Icebreaker | 15:10-15:15 | To start off the focus group discussion, I find it important to first get to know each other a little bit and also to exercise a little bit on how to use Mural. So that is why I want to start off with an icebreaker.  Add your name on your sticky note and place it next to the dog that best describes how you feel today.  Okay amazing, then we know how everybody is feeling today. |  |
| Ground rules | 15:10-15:15 | Because SRHR can be a sensitive topic, I first want to discuss some ground rules with you  Respectful Communication: Allow everyone to speak without interruptions. Listen actively and respect differing opinions, even if you do not agree.  Equal Participation: Everyone’s input is valuable. Please ensure that all voices are heard by allowing space for others to contribute.  Confidentiality: Respect the privacy of all participants. What is shared in this discussion should not be discussed outside the group without consent.  Do you all agree with this or do you have something to add? |  |
| Preliminary results action to knowledge framework | 15:15-15:30 | So then to start off the Focus group discussion, we will first discuss the preliminary results and do a member check.  So I will discuss the preliminary results in three parts. First I will go through the knowledge to action framework, then Challenges and opportunities in CoP´s and then the contextual factors. After each part I will ask you if this data resonates with your experience. If not, please put a red sticky note on the data you do not agree with  If you miss something, put your own colour sticky note on the slide with your idea  So when I am talking about the data, please keep this in mind that you have to do this.  Does anybody have any questions?  During the interviews I asked you about knowledge translation and the questions I asked were based on the knowledge to action framework. So this framework explains the processes behind knowledge translation. As you see it is not a step by step model, but it all interacts with each other.  Besides the model you see sticky notes. These sticky notes are either dark green or light green. The dark green sticky notes where things that were mentioned often and the light green sticky notes were things that were mentioned less.  Present the data  Does this data resonate with your experiences?   - If not, please put a red sticky note on the data you do not agree with - If you miss something, put your own colour sticky note on the slide with your idea   You have 5 minutes to complete this.  Does anybody have any questions?  Does anybody want to explain their sticky notes? |  |
| Preliminary results - challenges and opp | 15:30-15:45 | Present the data  Does this data resonate with your experiences?   - If not, please put a red sticky note on the data you do not agree with - If you miss something, put your own colour sticky note on the slide with your idea   You have 5 minutes to complete this.  Does anybody have any questions?  Does anybody want to explain their sticky notes? |  |
| Preliminary results - contextual factors | 15:45- 16:00 | Present the data  Does this data resonate with your experiences?   - If not, please put a red sticky note on the data you do not agree with - If you miss something, put your own colour sticky note on the slide with your idea   You have 5 minutes to complete this  Does anybody have any questions?  Does anybody want to explain their sticky notes? |  |
| Assignment challenges | 16:00-16:30 | So now we did the member check, we will go to page 2.1.1. This will be the first assignment.  For each challenge, what are some potential solutions or strategies that you think could help overcome this challenge?  Put sticky notes under each challenges with your ideas  You have got 10 minutes for this. After that we will discuss each challenge one by one.  Does anybody have any questions?  Does anybody want to elaborate their sticky note? Or does |  |
| Assignment contextual factors | 16:30-16:50 | Now we will go to assignment 3.1.1. This assignment is about the contextual factors. The first question is voting.  What 3 factors are most contextual factor that have an influence on knowledge translational activities are most prominent in your country?  Put sticky notes with the three contextual factors (from above) that are most prominent in your country in your box on this slide.  Does anybody have any questions?  Considering the contextual challenges we've identified, what strategies or approaches have you found effective in navigating these influences? Are there specific adjustments you've made to your knowledge translation practices?  Put a sticky note on this slide with your idea(s)  You have 7 minutes for this.  Then we will have a discussion about this (10 min) |  |
| Assignment CoP | 16:50-16:55 | So then the last assignment 4.2.  Do you believe that Communities of Practice (CoPs) are a valuable mechanism for knowledge translation? Why or why not?  Put a sticky note in the field you agree with and your reason for that answer.  If time, discuss this. |  |
| Ending | 16:55-17:00 | Is there anything else you would like to add?  Then I want to thank everybody for their valuable time and input. I really appreciate that you were all here today.  If you have any questions after this session please feel free to email me. I will also share the main findings of this focus group discussion with you asap and the draft article when it is finished.  If you have any questions after the Focus group discussion please let me know. Also if you felt unsafe at any point or if you feel like you said stuff that is unsafe for me to use. Let me know.  Then have a pleasant day. |  |

***Supplement 3:*** *Table internal challenges and opportunities of a CoP: codes, themes and descriptions*

| **Theme challenges** | **Code** | **Description** |
| --- | --- | --- |
| Structural and operational | *Lack of facilitation* | Absence of facilitation to guide the CoP, coordinate efforts, and ensure strategic direction. |
|  | *Lack of M&E strategy* | Insufficient monitoring and evaluation frameworks to track progress or measure impact. |
|  | *Limited resources* | Facing constraints in funding, time, and capacity, which hinder CoP activities. |
|  | *Limited institutional memory* | Experiencing a loss of knowledge due to member turnover or inadequate documentation of past activities. |
|  | *No clear goal* | Struggling with undefined or ambiguous objectives, making it difficult to align efforts. |
|  | *Magnitude of the problem* | Dealing with complex, large-scale issues that require extensive resources and long-term solutions. |
| Strategic positioning and influence | *Building reputation* | Challenges in building credibility and gaining recognition of Share-Nets role within the broader SRHR field. |
|  | *(Competition) Different organizations* | Balancing collaboration with competition among diverse organizations, which can create tension and conflict. |
|  | *Lack of dissemination strategy* | Struggling to effectively share knowledge products with stakeholders and reach the intended audience. |
|  | *Limited power* | Having minimal influence or authority to drive systemic change, particularly in policy contexts. |
| Relational and participation dynamics | *Different needs of members* | Challenges in addressing diverse and sometimes conflicting priorities among CoP members. |
|  | *Member engagement* | Facing challenges in maintaining active participation and consistent involvement from members. |
|  | *Voluntary participation* | Relying on voluntary participation, which affects consistency, commitment, and task completion. |
|  | *Being in a safe bubble* | Risk of remaining within a closed circle, limiting engagement with external perspectives and broader audiences. |
| **Theme opportunity** | **Code** | **Description** |
| Knowledge diversity and shared expertise | *Multidisciplinary* | Expertise from various fields contributes to holistic problem-solving and innovative approaches within the CoP. |
|  | *Repository of collective knowledge* | The CoP serves as a repository of collective knowledge, offering a valuable resource for addressing shared challenges. |
|  | *Geographic diversity* | The CoP benefits from members’ geographic diversity, providing a comprehensive understanding of what is happening across the country. |
|  | *International network* | Connections with organizations outside the country enhance cross-learning and provide opportunities to exchange diverse perspectives. (e.g. through International CoPs: i-CoP |
|  | *Sharing knowledge* | The CoP facilitates the exchange of ideas, expertise, and experiences, promoting learning and collaboration among members. |
| Supportive and motivating social environment | *Safe space* | The CoP provides an inclusive and supportive environment where members feel comfortable sharing ideas and engaging openly. |
|  | *Passion* | Members’ dedication and enthusiasm for the CoP’s mission drive sustained efforts and inspire impactful initiatives. |
|  | *Shared goal* | Members are united by a common vision and shared values, driving collective action toward meaningful outcomes. |
|  | *Social network* | Members leverage their social networks to provide emotional and social support. |
| Strategic positioning, visibility, and influence | *Avoid duplication of work* | CoP members gain insight into activities across different organizations, reducing redundant efforts and promoting efficient resource use. |
|  | *Independence* | Members enjoy the autonomy to make decisions and take initiative within the CoP. |
|  | *Powerful voices* | Influential members use their external power to support the CoP in achieving its goals and increasing its impact. |
|  | *Visibility* | The CoP builds its reputation and gains visibility, increasing recognition of its work and contributions. |
|  | *Uniqueness* | The CoP’s distinctive features set it apart as a unique platform. |
|  | *Benefit of Share-Net’s resources* | Share-Net’s resources and networks enhance the CoP’s capacity for learning, collaboration, and broader impact. |

***Supplement 4:*** *Contextual factor influencing KT process: codes, themes and descriptions*

| **Contextual Factor** | **Description** |
| --- | --- |
| *Acceptance and progressive* | Degree of societal openness and progressive attitudes that facilitate or hinder KT. |
| *Accessibility of SRHR services and education* | Availability and reach of sexual and reproductive health and rights information, services and education. |
| *Activism and movements* | Presence or absence of movements that support SRHR or other social and human rights. |
| *Being blocked or censored online as well as off-line* | Restrictions on (digital) platforms or content that limit the dissemination and sharing of knowledge. |
| *Capital loss* | Loss of infrastructure or resources reducing the ability to translate knowledge into practice. |
| *Catalyzing change* | External events or factors that accelerate shifts in attitudes, policies, or practices relevant to KT. |
| *Climate change* | Climate change leads to environmental challenges such as flooding and heat waves, which can disrupt KT processes and shift focus away from other important priorities. |
| *Colonialism* | Historical and ongoing impacts of colonial legacies that affect power dynamics, knowledge systems, and policy translation. |
| *Conservatism* | Conservative social, religion-interpretation inspired or political views that resist the sharing and implementation of translated knowledge. |
| *Corruption* | Presence of corruption that undermines KT processes. |
| *COVID* | The global pandemic’s impact on the ability to implement KT activities, including restricted access and resources. |
| *Culture* | Cultural norms and values that influence how knowledge is perceived, accepted, and applied. |
| *Difficulties in conducting research* | Challenges such as limited data, restrictive environments, or lack of access to populations that hinder knowledge production and application. |
| *Generation gap* | Differences in perspectives and approaches between age groups that affect collaboration and the acceptance of new knowledge. |
| *Government* | Support or opposition from government policies that influence the success of KT strategies. |
| *Language* | Language and literacy-level barriers and differences in terminology that create challenges in sharing and understanding knowledge. |
| *Law* | Legal frameworks that either support or restrict the implementation of translated knowledge. |
| *Gaps in awareness* | Gaps in awareness or understanding of key issues that slow down or obstruct effective KT. |
| *Not a priority* | Competing societal or governmental issues that deprioritize KT efforts. |
| *Patriarchy* | Gender inequalities that affect the dissemination and implementation of knowledge. |
| *Policies* | Existing policies that either facilitate or block KT processes. |
| *Police* | Law enforcement actions that may protect or suppress KT efforts. |
| *Politics* | Political dynamics that create opportunities or barriers to KT. |
| *Power imbalance* | Unequal power relations that hinder collaborative knowledge sharing or equitable application. |
| *Religion* | Religious beliefs that may align with or oppose the goals of KT initiatives. |
| *Rural areas* | Geographic remoteness that limits access to translated knowledge and resources. |
| *Safety and fear* | Concerns over personal safety that deter individuals from engaging in KT activities. |
| *Taboo* | Cultural sensitivities that restrict open dialogue and knowledge sharing on certain topics. |

***Supplement 5:*** *Table strategies to address the challenges: codes and descriptions*

| **Theme** | **Code** | **Description** |
| --- | --- | --- |
| **Adaptation to context** | *Access to knowledge* | Ensuring equitable knowledge access, especially for marginalized groups. |
|  | *Context is key* | Adapting activities to local contexts and documenting these insights for implementation. |
|  | *Cross-learning* | Encouraging knowledge exchange within Share-Net and with external organizations. |
|  | *Long-term initiatives* | Sustaining CoP initiatives for long-term impact. |
|  | *Stakeholder involvement* | Engaging stakeholders early to ensure inclusivity. |
|  | *Strategic messaging* | Crafting logical, well-planned messages to engage diverse audiences. |
| **Dissemination** | *Accessibility and inclusivity* | Ensuring knowledge is accessible to diverse groups through appropriate language, media, and formats. |
|  | *Dissemination strategy* | Developing tailored plans to reach end users. |
|  | *External dissemination* | Sharing knowledge via Knowledge Fairs, local media, CoP members and external platforms. |
|  | *Internal dissemination* | Promoting cross-learning within Share-Net’s CoPs and among members. |
|  | *International network* | Collaborating across CoPs in different countries to work towards shared goals and learn from each other. |
|  | *Policy makers engagements* | Critically engaging governments and addressing gaps in implementation. |
| **Monitoring & evaluation (M&E)** | *Baseline* | Establishing baselines to measure the long-term impact of CoP initiatives. |
|  | *CoP on M&E* | A special CoP designed to provide M&E. |
|  | *Feedback mechanisms* | Implementing surveys and forums to gather community input and identify needs. |
|  | *Driven by internal needs* | Conducting M&E for internal growth and learning, beyond external reporting requirements. |
|  | *Standard framework* | Creating a standard framework to track progress, align possibly with SDGs, and ensure follow-up. |
| **Member engagement** | *Advertising membership* | Promoting membership internally and through strategic outreach. |
|  | *Appreciation and respect* | Recognizing and celebrating contributions from members and encouraging mutual respect. |
|  | *Clear benefits* | Highlighting tangible benefits of being a CoP member such as training, grants, and networking opportunities. |
|  | *Clear goals* | Linking activities to external goals or events helps ensure efficient KT. |
|  | *CoP shelf life* | Ensuring the sustainability and relevance of CoPs over time, to decide to stop as CoP if no longer relevant or needed. |
|  | *In-person meetings* | Encouraging face-to-face engagement to strengthen collaboration. |
|  | *Internal member motivation* | Acting on the desire and needs of the members. |
|  | *Knowledge within the CoP* | Increasing the knowledge within a CoP on knowledge translational processes. |
|  | *Ongoing engagement* | Regularly updating members, inviting them to events, and ensuring their involvement. |
|  | *Strengthen KT* | Improving processes for translating knowledge into actionable outcomes. |
|  | *Passion* | Highlighting that absence of financial resources should not deter activity. |
| **Resources** | *Co-creation* | Partnering to optimize resource use and efficiency. |
|  | *Fundraising strategy* | Securing diverse funding through paid services, local funds, and private-sector partnerships. |
|  | *Resource management* | Efficiently managing resources without compromising CoP objectives. |
|  | *Funding for M&E* | Integrating funding to support effective M&E activities. |
|  | *Facilitation role of Share-Net* | Strengthening KT processes and member motivation through a designated person of Share-Net. |
